# Color Maps: Facilitating the Clinical Impact of Quantitative MRI

**DOI:** 10.1002/jmri.29573

**Published:** 2024-08-23

**Authors:** Nico Sollmann, Miha Fuderer, Fabio Crameri, Sebastian Weingärtner, Bettina Baeßler, Vikas Gulani, Kathryn E. Keenan, Stefano Mandija, Xavier Golay, Nandita M. deSouza

**Affiliations:** ^1^ Department of Diagnostic and Interventional Radiology University Hospital Ulm Ulm Germany; ^2^ Department of Diagnostic and Interventional Neuroradiology, School of Medicine, Klinikum rechts der Isar Technical University of Munich Munich Germany; ^3^ TUM‐Neuroimaging Center, Klinikum rechts der Isar Technical University of Munich Munich Germany; ^4^ Radiotherapy, Division Imaging and Oncology University Medical Center Utrecht Utrecht The Netherlands; ^5^ Undertone.design Bern Switzerland; ^6^ Department of Imaging Physics Delft University of Technology Delft The Netherlands; ^7^ Department of Diagnostic and Interventional Radiology University Hospital Wuerzburg Wuerzburg Germany; ^8^ Department of Radiology University of Michigan Ann Arbor Michigan USA; ^9^ Physical Measurement Laboratory National Institute of Standards and Technology Boulder Colorado USA; ^10^ Queen Square Institute of Neurology University College London London UK; ^11^ Gold Standard Phantoms Sheffield UK; ^12^ Bioxydyn Manchester UK; ^13^ The Institute of Cancer Research London UK; ^14^ The Royal Marsden NHS Foundation Trust London UK

**Keywords:** color maps, relaxometry, diffusion, contrast‐enhanced imaging, elastography, fat fraction

## Abstract

Presenting quantitative data using non‐standardized color maps potentially results in unrecognized misinterpretation of data. Clinically meaningful color maps should intuitively and inclusively represent data without misleading interpretation. Uniformity of the color gradient for color maps is critically important. Maximal color and lightness contrast, readability for color vision‐impaired individuals, and recognizability of the color scheme are highly desirable features. This article describes the use of color maps in five key quantitative MRI techniques: relaxometry, diffusion‐weighted imaging (DWI), dynamic contrast‐enhanced (DCE)‐MRI, MR elastography (MRE), and water‐fat MRI. Current display practice of color maps is reviewed and shortcomings against desirable features are highlighted.

**Evidence Level:**

5

**Technical Efficacy:**

Stage 2

Medical images are routinely represented in grayscale and interpreted by subjective visual assessment of the depicted anatomy and any pathological variation. Increasingly, imaging techniques from a variety of modalities (computed tomography [CT], MRI, and positron emission tomography [PET]) allow additional quantitative data to be derived, relating to underlying tissue characteristics such as cell density, tissue stiffness, vascularity, and metabolism. Color coding of such quantitative information overlaid on the anatomic images has become widely adopted. The most common example is from hybrid imaging techniques such as PET/CT. Here, the standardized uptake value (SUV) of an ^18^F‐fluoro‐2‐deoxy‐glucose (FDG) tracer representing tissue glucose metabolism is overlaid on the grayscale anatomic CT scan constituted from tissue attenuation of incident X‐rays. Rather than standardize the color display itself, and because only one metric (SUV) is quantified, a program of scanner accreditation through initiatives such as the European Association of Nuclear Medicine Research Ltd. (EARL) is used.^1^, ^2^, ^3^ Normalization of the SUV to a background value in accredited scanners has resulted in a widely adopted and clinically acceptable approach to harmonization strategies in PET quantification with related color maps.^1^, ^2^, ^3^

Application of MRI is characterized by its versatility in deriving multiple tissue characteristics within a single examination. Multiple parameters such as tissue relaxation times (eg, T1, T2), cell density (diffusion‐weighted imaging [DWI]), vascularity (dynamic contrast‐enhanced MRI [DCE‐MRI]), metabolism (MR spectroscopy [MRS]), stiffness (MR elastography [MRE]), macromolecular content (chemical exchange saturation transfer [CEST] and amide proton transfer [APT]), and proton density fat fraction (PDFF from water‐fat imaging) can be quantified. Multi‐parametric color maps are used to depict parameter distributions in relation to each other within the underlying anatomy. In the brain, multi‐parametric maps have been used to probe tissue microstructure,^4^ in cancer imaging they have been used for tumor detection and grading,^5^ in cardiac MRI to depict left ventricular function,^6^ and in musculoskeletal imaging to visualize longitudinal changes in cartilage.^7^ Unfortunately, despite visual appeal for image interpretation, there has been a surprising lack of standardization in the development of color maps, making comparisons across studies and institutions difficult and sometimes misleading.

To initiate the process of standardization of color maps, and in parallel with conducting a survey according to the Delphi method to achieve a consensus for color representation of MR relaxometry data,^8^, ^9^ this article aimed to outline the considerations when standardizing color maps, highlighting desirable features, and describing the clinical use of color maps and their current conformance to these desirable features. Color maps used for five key quantitative MRI techniques are discussed: relaxometry, DWI, DCE‐MRI, MRE, and water‐fat imaging as they represent the common clinically used methods for obtaining quantitative parameters in MRI.

## Considerations When Standardizing Quantitative Imaging Biomarker Color Maps—Desirable Features

**FIGURE 1:**
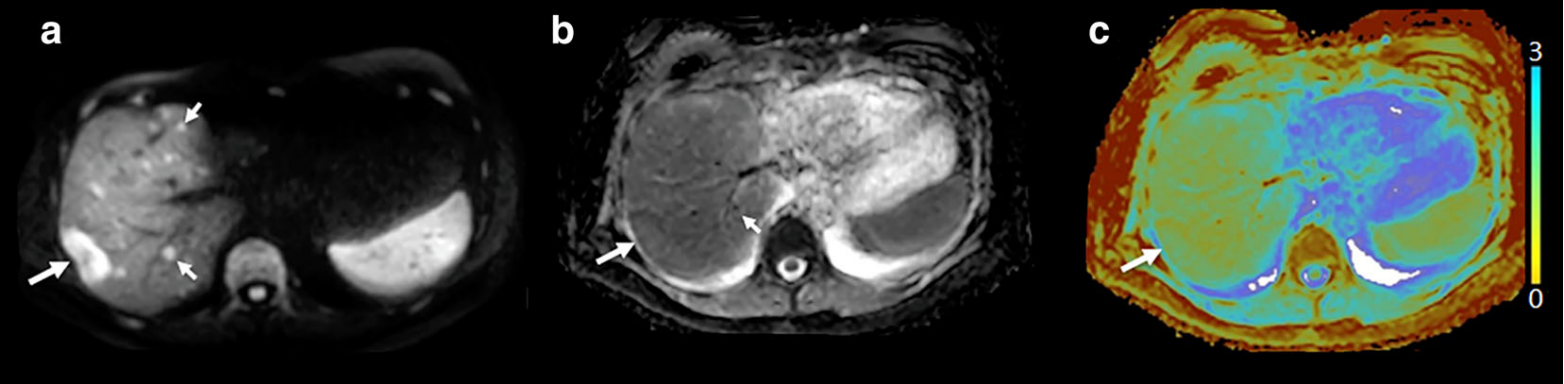
Liver metastases from neuroendocrine cancer in the pancreatic tail on diffusion‐weighted imaging (DWI, *b* = 800 s/mm^2^) (**a**), apparent diffusion coefficient (ADC) map in grayscale (**b**), and depicted in a color map generated from quantitative ADC data using a commercially available software platform (**c**), indicating the importance of dynamic range in depicting the disease on a color map. Although the large (big arrow) and numerous small metastases (small arrows) are well seen on the signal intensity images (**a**), they are much less obvious in (**b**) where there is a small but appreciable difference in ADC compared to normal liver (0.88 ± 0.08 mm^2^/s for tumor, 1.33 ± 0.11 mm^2^/s for normal liver), and are virtually invisible in (**c**) when a non‐optimized color map is applied across the entire range of measured ADC, without adjusting for the displayed dynamic range.

Presenting quantitative data using non‐standardized color maps potentially results in unrecognized misinterpretation of data (eg, by highlighting some data points over others). For instance, a lack of uniformity of the color gradient can over‐ or under‐represent the true disease status (Fig. 1). Unfortunately, although non‐standardized color representation of quantitative parameters is the norm, no formal data exist on the extent of misinterpretation that results in clinical practice. The rainbow or jet color palettes, which are the most widely known and applied and often included as the default palettes by commonly used software, can be limiting.^10^ Yellow, the brightest and thus most attractive color to the human eye, is not at any end of the color range in the rainbow spectrum.^10^, ^11^ Hence, in the clinical setting, this may lead to emphasizing one site of the disease over another, or could result in even obscuring the disease when an inappropriate dynamic range is applied (Fig. 1). Moreover, a combination of specifically red and green is problematic for those with red‐green weakness, a very common color vision deficiency.^10^, ^12^, ^13^

**FIGURE 2:**
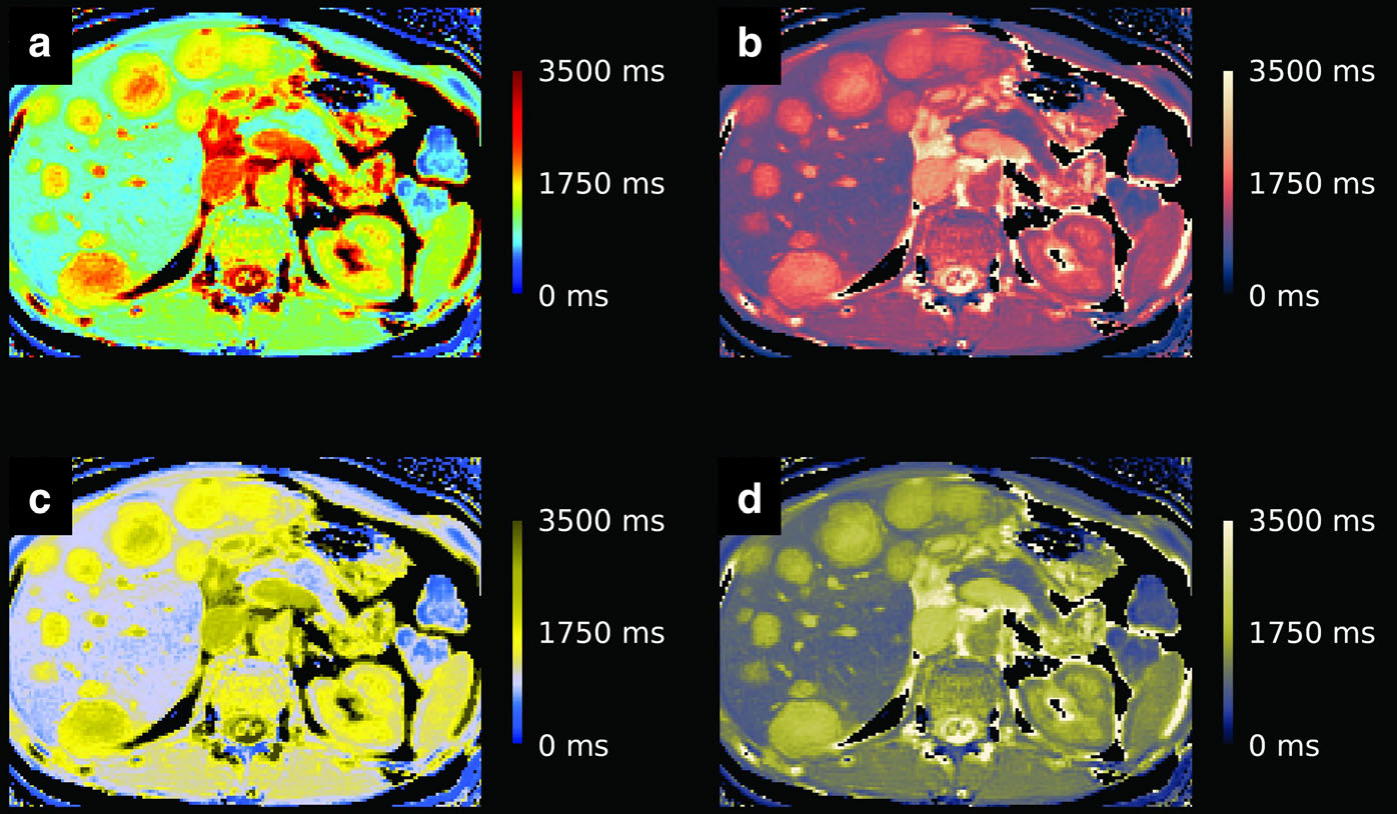
T1 color maps through the liver with multiple metastatic lesions using the jet scheme (**a**) and using the Lipari scheme (**b**).^77^, ^78^ The contrast along the color gradient is non‐uniform in (**a**) compared to (**b**), and the magnitude of the color gradient is non‐constant, leading to false interpretation of the quantitative metrics. Yet, the factors are improved in (**b**). Simulations of the jet color map image (**c**) and the Lipari color map image (**d**) as seen by persons with deuteranopia, the most common type of (“red‐green”) color vision impairment. The color spectrum as displayed in (**c**) shows the most luminant part in the center rather than at the top of the range, while in (**d**) the most luminant part remains represented at the top of the range. The jet color palette is widely used, but fails to adhere to the major desirable features (ie, even color contrast along the color gradient for accurate representation of data, high overall lightness contrast of the color gradient, intuitive and constant magnitude of the color gradient along the whole color scale, and recognizability of the type of information that is presented).

A color map should intuitively and inclusively represent the data without misleading interpretation. Perceptual uniformity, representing constant color gradients all along the color range, is a crucial feature. A constant color gradient means that each step of the range has a constant distance in the L*a*b* color space (where “*L*” represents luminance and “*a*” and “*b*” are two chrominance coordinates).^14^ A major feature of a desirable color map is perceptual color order, guaranteeing that the gradient of color is intuitively understandable.^10^ Other major aspects should be universal readability (eg, by individuals with color vision deficiencies), and most importantly, color palettes should effectively convey the underlying data and their nature (Fig. 2).^10^ Biomarkers with linear scales (eg, shear modulus for MRE) can be more easily standardized. Previous work has suggested optimal color map designs for the research community, and some options for suggested maps have been made freely available,^15^, ^16^, ^17^, ^18^ but still lack consensus and widespread adoption. Hence, the following major desirable features of a standardized color map may be recognized (Table 1)^10^, ^16^, ^17^, ^18^, ^19^, ^20^, ^21^:

1. Even color contrast along the color gradient for accurate representation of data (only a perceptually uniform color map weights the same data variation equally across the data space and limits adding visual errors to the data).
2. High overall color contrast of the color gradient (ensuring good visibility of imaged features in the data for people with normal color vision).
3. High overall lightness contrast of the color gradient (ensuring universal and good visibility of imaged features in the data also for people with impaired color vision).
4. Intuitive and constant magnitude of the color gradient along the whole color scale (a perceptually ordered color map does not break the correspondence between color and numeric values and enables qualitative understanding of a dataset).
5. Recognizability, from the color scheme, of the type of information that is presented (by fostering a uniform, coordinated use of individual color schemes for given types of data not only over many users, institutes, countries, and MRI manufacturers, but also over time).

**TABLE 1:** List of desirable features of a standardized color map in MRI

1) Even color contrast along the color gradient
2) High overall color contrast of the color gradient
3) High overall lightness contrast of the color gradient
4) Intuitive and constant magnitude of the color gradient
5) Recognizability

## Clinically Used Quantitative MRI Biomarkers Represented by Color Maps—Conformance With Desirable Features

### Relaxometry

**FIGURE 3:**
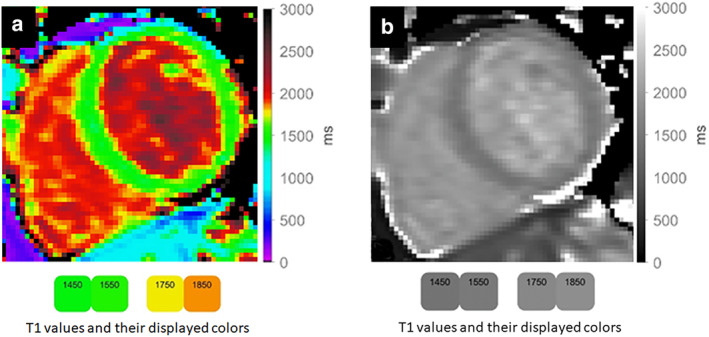
Myocardial T1 map (**a**) and corresponding grayscale image (**b**) acquired at 3 T and displayed with a visually non‐linear color map.^23^ The color map was designed to be perceptually insensitive around the normal reference values (~1500 ms). The color bars illustrate that 100 ms difference between 1450 ms and 1550 ms are visually not discernable, while a clear difference is discernable for a difference of 100 ms between 1750 ms and 1850 ms.

Relaxation times describing the temporal evolution of the MRI signal have been identified as useful biomarkers to distinguish health and disease (Figs. 2 and 3).^22^, ^23^ In addition to T1 and T2, many time constants have been proposed to describe the signal evolution more accurately, more specifically, or for certain circumstances such as stimulated recovery. For example, prolonged T2 times have been used to indicate tissue edema,^24^, ^25^ shortened T2* times the presence of iron deposition,^26^, ^27^ and shortened T1 times fatty infiltration.^28^, ^29^

Default color maps of relaxometry from scientific programming environments such as Matlab or Python, or color maps borrowed from other imaging modalities such as PET/single‐photon emission CT (SPECT),^30^, ^31^ are spatially resolved and are assessed qualitatively or quantitatively for the whole organ, segmental regions, or only in regions of interest. Additional processing allows the normal range to yield no visual color contrast, with discernible contrast only in the presence of pathological alterations. In inflammation studies, T1 relaxation values from 0 up to 5000 ms have been represented on linear scales (Fig. 3),^32^ and similar arbitrary scales have been applied for T2* relaxometry in uterine applications.^33^ Standardized protocols for creating a T2* map with scanner‐independent software have recently been attempted,^34^ but again these fail to adequately embrace the appropriate visual scale and the ranges of change that need to be depicted to the interpreter.

Usage of rainbow, jet, heat, and other general color map palettes is widespread. In previous work, the jet color map was used for all types of quantitative relaxation metrics.^7^ Since both jet and rainbow palettes fail the major desirable features as outlined above, the whole set that is representative for the usage of color in relaxation image display fails the major desirable features (1 and 3–5 of the listed features above).

### Diffusion‐Weighted MRI

**FIGURE 4:**
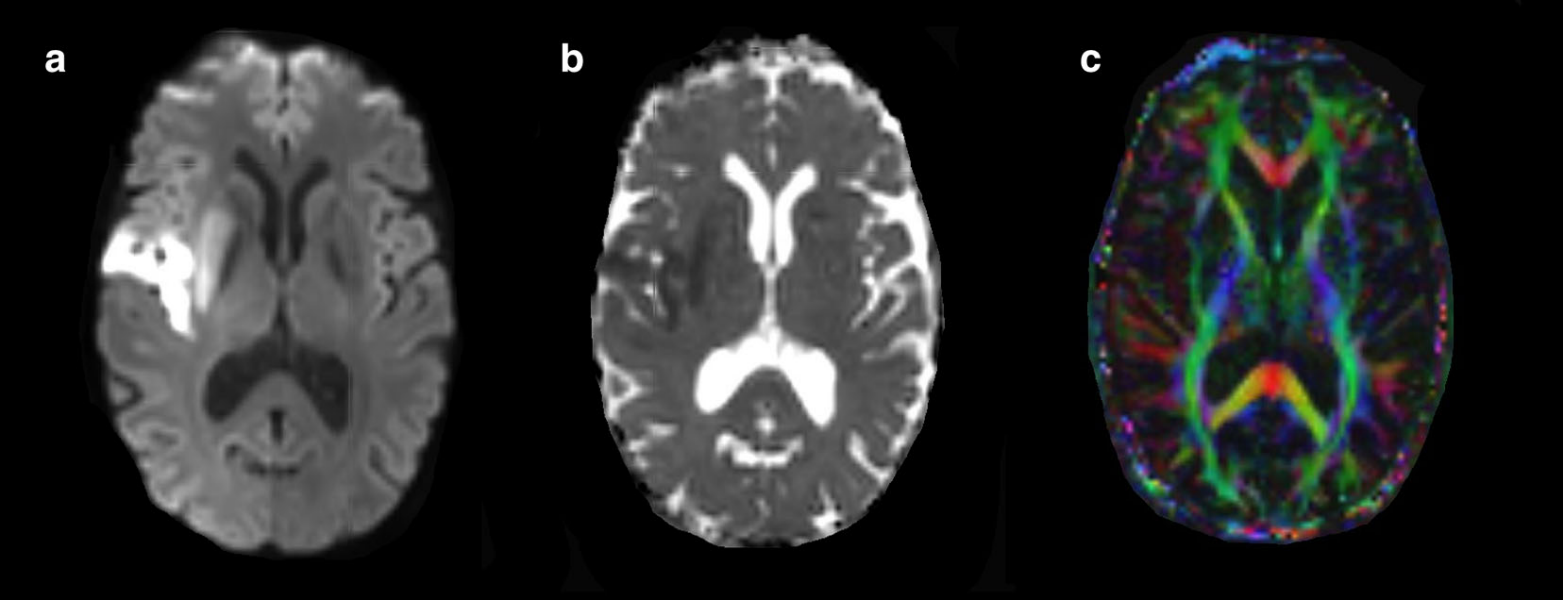
Diffusion‐weighted imaging (DWI) of the brain (**a**) with the corresponding apparent diffusion coefficient (ADC) map (**b**) and diffusion tensor imaging (DTI) map (**c**). Restricted diffusion due to right‐hemispheric ischemic stroke (affecting the insular region as well as putamen) in (**a**) is visualized as hyperintense signal. Corresponding ADC signal decreases in (**b**) are shown in grayscale. In (**c**), the orientation of white matter fiber bundles is shown within the brain by assigning a principal color component (red, green, and blue) to a principal direction (left–right, anterior–posterior, and superior–inferior), referred to as directionally encoded color (DEC). Although widely used, good visibility for people with impaired color vision is difficult for DTI data with DEC, given that it needs three color coordinates to convey information on a three‐dimensional vector.

Apparent diffusion coefficient (ADC) mapping, which reflects water self‐diffusivity in tissues,^35^, ^36^, ^37^ is widely used in diverse applications including stroke imaging (Fig. 4),^38^ white matter tract mapping in the brain,^39^ and oncological imaging throughout the body.^40^ Although ADC maps in clinical practice are largely displayed in grayscale, color‐coded maps of ADC in the brain have been sometimes utilized with different arbitrary ranges of values, selected to highlight contrasts in the various compartments (gray matter, white matter, and cerebrospinal fluid).^41^

Particularly in white matter, diffusion may be anisotropic. Mathematically, this is described by a second‐order tensor; the ratio between the eigenvalues thereof indicates the level of anisotropy and the principal eigenvector indicates the orientation of fibers. The degree of anisotropy in each voxel, such as fractional anisotropy (FA) or relative anisotropy, is a scalar metric that can be displayed using a grayscale or color map. The orientation is usually displayed by assigning a principal color component (red, green, and blue) to a principal direction (left–right, anterior–posterior, and superior–inferior), referred to as directionally encoded color (DEC).^42^ The intensity of such a color‐coded image can be made dependent on the FA.^43^, ^44^, ^45^ In addition, multiple display schemes are used to follow and display white matter tracts in the brain (tractography), where a single arbitrarily selected color is used to label a particular fiber tract.^39^, ^46^ The position and orientation of fiber tracts can be used to show their invasion and displacement by tumors,^47^, ^48^ prior to surgical planning for brain tumors,^42^, ^49^, ^50^ and for post‐surgical follow‐up examinations.^51^, ^52^

The ADC values are commonly displayed in grayscale with only minor exceptions. Currently, DEC maps employ a standardized color scheme,^42^ and the type of image is clearly recognizable. Unfortunately, good visibility for people with impaired color vision is difficult for DEC, since it needs three color coordinates to convey information on a three‐dimensional vector. However, DEC fulfils as many of the desirable properties of color maps as possible. Nevertheless, there is no single accepted display standard for FA metrics.

### Dynamic Contrast‐Enhanced MRI

**FIGURE 5:**
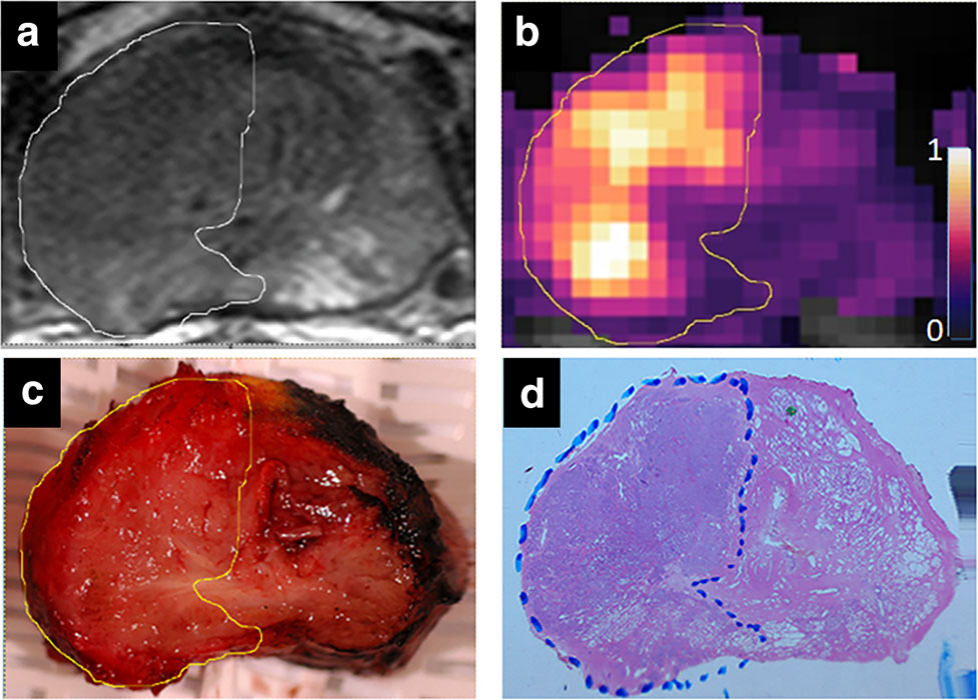
T2‐weighted image (**a**), volume transfer constant (K^trans^) color map (**b**), macroscopic fresh slice following prostatectomy (**c**), and whole mount histology (**d**) for comparison showing right‐sided prostate cancer (segmented in yellow outline). The K^trans^ (min^−1^) color map is shown in an arbitrary palette color scale from purple to white with maximum and minimum values represented at the ends of the scale. Variations in K^trans^ are discernible across the tumor that may be linked to tumor biology.

Imaging by DCE‐MRI is used to evaluate blood flow and perfusion within an organ. Color displays dependent on the increase in signal intensity following administered contrast agent are typically generated for an individual case series.^53^, ^54^ The pharmacokinetic parameters (eg, volume transfer constant [Ktrans], initial area under gadolinium curve [IAUGC], relative cerebral blood volume [rCBV], mean blood flow [MBF], and many custom‐made or industry‐provided metrics) derived from mathematically modeling the acquired dynamic data are also processed to generate color maps that enhance the visual perception of heterogeneity in tissue perfusion and permeability (Fig. 5).

Early work commonly displayed color maps without a color scale to indicate what the colors represented, the default assumption being that blue represents a low value and red a high value in terms of perfusion.^55^ Color displays of brain tumor perfusion (rCBV) and permeability (K^trans^) showed color overlays of these parameters in green, yellow, and red (as per a traffic light system) to denote low, intermediate, and high values, often without defining these ranges.^56^ In other instances, a researcher‐selected color scale has been used to illustrate visually detectable changes in myocardial perfusion.^57^ Despite the increasing use of color to depict tissue perfusion over the last decade, the choice of scales and ranges remains arbitrary and applicable to individual purposes.^58^

The subjective interpretation of color‐coded maps for DCE‐MRI has been reported, and use of different color scales has influenced the perception of tumor heterogeneity and aggressiveness,^59^ as well as the assessment of perfusion parameters and lesion conspicuity in prostate cancer.^60^ Use of different color maps has also affected the diagnostic accuracy and confidence of radiologists in breast cancer,^61^ where a linear or a logarithmic grayscale is recommended instead. However, a sufficient sample size per DCE‐MRI metric (eg, rCBV or K^trans^) to support a statement on the uniformity of the color scale used has not been proposed. Given the widespread use of the jet color palette, it is likely that current practice at least partly fails on desirable color map features 1, 3, and 4.

### MR Elastography

Viscoelastic characteristics of tissue based on the propagation of mechanical waves generated by a dedicated actuator can be evaluated by MRE.^62^ Specifically, MRE has been primarily developed for liver applications,^63^ but breast,^64^ brain,^65^ and musculoskeletal applications^66^ are gaining traction. The calculated stiffness and shear modulus are displayed using a color map across the entire color spectrum with commonly violet at minimum and red at maximum. The color spectrum typically represents values from 0 kPa to 8 kPa in the liver^67^ and from 0 kPa to 5 kPa in the brain.^68^

In MRE, two types of images are often displayed in color: the wave image and the elastogram (or stiffness image). It is stiffness that is currently the parameter of interest in clinical usage. Although for stiffness, the use of the rainbow color palette seems to be dominant, other color maps also have been applied.^68^ As stated in relaxometry, the major desirable color map features 1 and 3–5 are not fulfilled. Moreover, changes in stiffness across a clinically relevant range may be unevenly distributed so that relevant changes may be over‐ or under‐represented. This makes images potentially susceptible to misinterpretation or difficult to compare between studies with different maximum values.

### Water‐Fat MRI

Quantitative assessment of fat within tissue may be evaluated by exploiting the resonance difference between the proton frequencies in water and fat.^69^ The estimated tissue fat content expressed as a percentage (PDFF) is the key metric derived for usage in both clinical and research settings for diagnosis and grading of severity of hepatic steatosis in a cross‐sectional design,^70^ or for monitoring response to treatment in longitudinal evaluations.^71^ As with MRE, color maps of PDFF are displayed using the entire visual color spectrum from violet to red in a linear manner to indicate a fat percentage from 0% to 100%. As normal liver fat percentages are less than 10%, and the increase in fat in hepatic steatosis is typically up to 40%,^72^ the color scale representation is not ideally sensitive to changes in PDFF in subjects with hepatic steatosis where the majority of the scale from 40% to 100% is not utilized. Moreover, when employed to assess fat composition in other organs such as muscle or bone^73^, ^74^, ^75^ and in paediatric populations^76^ where smaller changes in absolute value of fat content may need to be represented, the poor sensitivity of color maps displayed using such a scale may not add value to the numerical data.

For PDFF, we observe the use of rainbow, jet, and grayscale as the most common color palettes. As with relaxometry and MRE, the major desirable color map features 1 and 3–5 are not fulfilled.

## Conclusion

Apart from ADC and DEC for DWI, most quantitative MRI‐derived biomarkers fail most of the desirable properties of appropriate color display. Moreover, color maps specifically addressing the clinical usage of specific biomarkers from quantitative MRI have not been agreed upon, nor have there been specific guidelines or recommendations for standard use with clinical applications. The Color Recommendation Committee, which was launched from the Quantitative MRI Study Group of the International Society for Magnetic Resonance in Medicine (ISMRM), has completed a Delphi process to provide guidelines through consensus on the generation of color maps for MR relaxometry.^8^ Consensus was reached on the type of color maps for T1 and T2 with the mandatory use of color bars and the use of a specific color to indicate “invalidity” of a value.^8^ It is envisaged that this is a first step for achieving a more standardized approach for a range of quantitative MRI parameters in a range of clinical applications.
